# Emerging botulism in Vietnam: Identifiable risk factors, future perspective, and recommendations

**DOI:** 10.7189/jogh.14.03004

**Published:** 2024-01-12

**Authors:** Ngoc Ha Tran, Van Phu Tran, Dang Nguyen, Nham Tran

**Affiliations:** 1 Department of Otolaryngology, University of Medicine and Pharmacy at Ho Chi Minh City, Vietnam; 2 School of Biomedical Engineering, Faculty of Engineering and Information Technology, the University of Technology Sydney, Ultimo, New South Wales, Australia; 3 Tra Vinh University, Tra Vinh City, Vietnam; 4 Massachusetts General Hospital, Corrigan Minehan Heart Center, Harvard Medical School, Boston, Massachusetts, USA

**Figure:**
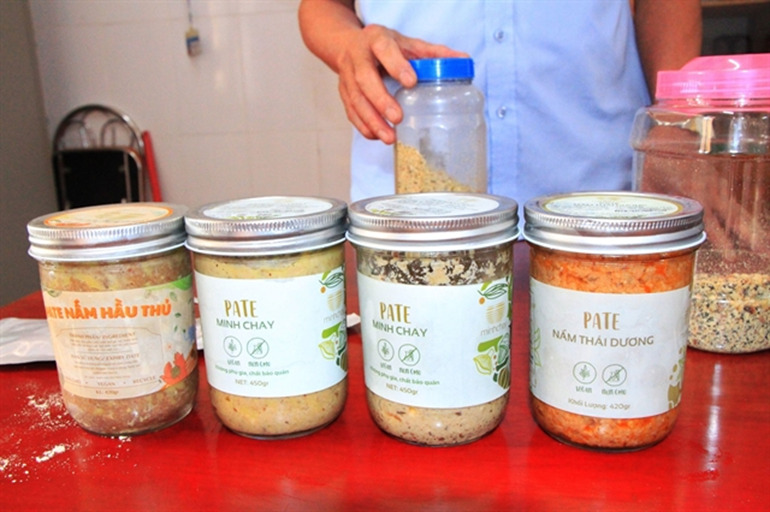
Photo: Products of Loi Song Moi Two members Ltd Company are found and withdrawn in the central province of Ha Tinh, free to use under CC BY 4.0 license. Available: https://vietnamnews.vn/society/772095/manually-canned-food-poses-risks-of-poisoning.html

The gram-positive, anaerobic bacterium *Clostridium botulinum* (*C. botulinum*) causes botulism, a severe neuroparalytic disease that affects both humans and vertebrate animals. This bacterium is typically found in the form of endospores in nature. Unless *C. botulinum* spores germinate and create vegetative cells that produce neurotoxins, people are frequently not at risk from it. Spores typically pass through our bodies and are excreted without any harm since a healthy human digestive system does not allow spore germination, with the exception of cases of newborn and adult gut dysbiosis botulism [1]. Due to its high potency and mortality, botulinum neurotoxin (BoNT) is regarded as the deadliest toxin with a lethal dose of 1–3 ng of toxin per kg of body mass. Botulism causes an irreversible suppression of acetylcholine (ACh) release at the presynaptic nerve terminal of the body's neuromuscular junctions, resulting in flaccid paralysis [2].

Since *C. botulinum* was first identified as a foodborne pathogen in Germany and Belgium in the 1800s and the first cases of botulism were tracked by the Centers for Disease Control and Prevention (CDC) in the United States in 1973 [2], there have been several occurrences of the disease since then. In July 2020, botulism infections were reported in Vietnam, more than 20 individuals became ill after consuming the infected food products and were treated in hospitals in Hanoi, Ho Chi Minh City, and Dong Nai Province. In these cases, *C. botulinum* type B bacteria was detected in the vegan pâté sold under the Minh Chay brand – a product of Loi Song Moi Two Members Ltd Company [3–5]. In March 2021, one person died of suspected botulinum poisoning after eating vegetarian pâté in Binh Duong province [6]. According to the Binh Duong Food Safety and Hygiene Department, further infections occurred in another 25–30 individuals. The first confirmed case of infant botulism occurred on 28 April 2021, in Hanoi, where a 10-month-old infant was infected by *C. botulinum* [7]. Recently, in May 2023, an alarming series of six additional patients have been infected in Ho Chi Minh City, resulting in one fatality [8]. The Vietnam Ministry of Health reported that these six patients had consumed bread with pork bologna sold by street vendors and another 45-year-old patient was hospitalised after consuming fermented fish sauce [9]. These recent occurrences mark the first confirmed cases of such a diagnosis in major Vietnamese cities, raising concerns about potential risks and the need for preventive measures.

## RISK FACTORS OF BOTULISM

There are three main kinds of botulism [1,2,10]. Foodborne botulism is the best-known type when foods containing low-acid preservation of vegetables, canned tuna, fermented, salted and smoked fish, as well as meat products like ham and sausage, have all been linked to botulinum toxin in different parts of the world [10]. The precise ingredients can differ between various countries, reflecting local dietary customs and methods of food preservation [10]. Infant botulism is considered the most common and may be one cause of sudden infant death. Most infants that are infected are less than six months of age [11]. Infantile botulism occurs when babies consume contaminated milk or food, the neurotoxin is absorbed in the large intestine. The most common cause of contaminated food and dust is the *C. botulinum* toxin. There is a substantial correlation between home-canned and preserved goods, improper sterilising methods, and inadequate refrigeration. About 20% of botulism cases involve honey or corn syrup [12]. Other sources of spores have originated from contaminated powdered baby formula and household dust [1]. Wound botulism a rare infection occurs when the bacterium enters an open wound and releases the neurotoxin. While the symptoms of foodborne botulism are similar, they may take up to two weeks to manifest. This delay may complicate diagnosis and requires careful clinical observation. The falling, flaccid paralysis that might result in respiratory failure is a hallmark of foodborne botulism. Vertigo, severe exhaustion, and weakness are among the early symptoms. These are typically followed by blurred vision, dry tongue and difficulty swallowing and speaking. There may also be nausea, diarrhea, constipation, and abdominal edema. The condition can advance to cause weakness in the neck and arms before affecting the respiratory muscles and lower body muscles [10]. Substance misuse has also been linked to this condition, especially when injecting black tar heroin [1,10].

## RISK FACTORS OF BOTULISM IN VIETNAM RELATED TO TRADITIONAL CULTURE AND HABITS

Due to poisoning from improperly stored food, botulism has been described as posing a particularly significant risk to humans in northern climate regions [13]. Research in the area of food safety has looked into spore resistance as well as the conditions that encourage and inhibit the growth of *C. botulinum* [13]. In cured meat products, nitrite has long been used to prevent *C. botulinum* growth. But in the case of dry fermented sausages, environmental elements such as pH and the competing microbiota might play a more significant effect than nitrite in preventing *C. botulinum* from growing and producing toxins [14].

Certain traditional Vietnamese meals and dishes, including fermented fish sauce, salted fish, and pickled vegetables, may pose a risk if improperly made, stored, or processed. Additionally, consuming contaminated canned or bottled foods can also lead to botulism. The warm tropical weather in Vietnam poses an additional risk, as improper storage or leaving food at room temperature for extended periods can promote bacterial growth and toxin production. Pathogens like *Clostridium botulinum, Salmonella sp, Listeria sp* and *Staphylococcus aureus* were reported from many fermented fish products [15]. In Vietnam, fermentation practices can pose health risks if not conducted in accordance with proper hygiene and food preparation standards. Recent infections of *C. botulinum* in the country were traced to various food products, including vegetarian pâté, honey, pork bologna, and fermented fish sauce. This highlights the necessity for stringent oversight and adherence to food safety protocols, especially in the preparation and storage of fermented food products. The presence of *C. botulinum* in these widely consumed items underscores the vital importance of educating food producers and the general public on safe fermentation techniques, to mitigate the risk of contamination.

## FUTURE PERSPECTIVE AND RECOMMENDATIONS

Botulism occurrences can have severe consequences, given the large consumption of these food products in the local area, disease management plans must be implemented to prevent the spread of this bacterium. We proposed several recommendations on how to handle the occurrences, emphasising early detection, medical intervention, preventive measures, and possible community management. By following these guidelines, we can increase our ability to respond effectively to further outbreaks, and mitigate the risk of contamination to ensure public health.

### Botulism Antitoxin Heptavalent

All previous non-infant botulinum antitoxins were replaced as of 13 March 2010, by the Heptavalent Botulinum Antitoxin (BAT) [16]. Emergent BioSolutions Canada Inc. (EBCI) (Winnipeg, Canada) is the manufacturer and license holder of the BAT product [16]. For adult and pediatric patients with symptoms of foodborne or wound botulism or probable exposure to botulinum toxin A–G, the FDA has approved BAT [17]. In individuals who received treatment, BAT was secure and beneficial clinically. Shorter hospital and intensive care stays were linked to the administration of BAT within two days of symptoms [16,18]. These findings emphasise the significance of retaining a clinical suspicion for botulism in patients presenting with paralytic disease in order to enable prompt BAT treatment before possible test confirmation [19].

Of concern is that the supply of BAT required for treating botulinum poisoning is limited worldwide, which is an added challenge for early treatment measures [16]. Moreover, the cost of BAT is very high, and it is not covered by health insurance in Vietnam. According to a study conducted by Deborah M Anderson and colleagues, patients who received BAT treatment early compared to late had a total mean cost per patient that was 2.5 times lower (USD 37 607 compared to USD 94 223 for late treatment) and early BAT product treatment, as opposed to late treatment, could save more than USD 3.9 million annually on average in the US [18]. During the 2020 botulism outbreak in Vietnam, a total of thirteen cases were reported. The financial cost associated with the treatment of this illness was significant. The estimated per-patient cost for treatment in a non-intensive care unit (ICU) setting was USD 18 506, while the cost for those requiring ICU care was USD 415 576 and mechanical ventilation (MV) reached USD 774 572. These expenses added up to a total of USD 1 208 654 per patient. The high costs underscore the economic impact of the disease and highlight the importance of early intervention [20].

At present, efforts are being made by the Drug Administration of Vietnam to procure the necessary drugs through discussions with the World Health Organization (WHO) [8]. However, in recent cases of botulism in May 2023, a distressing incident occurred when one botulinum patient died shortly after the arrival of six vials of BAT from the WHO's warehouse in Switzerland [8]. In this specific case, the best choice is to use the antidote, but this must be done at the first sign of weakness to let the antidote neutralise toxins. The BAT antidote, provided by WHO, had already arrived at the hospital, but the patient had missed the golden period for taking the medication. The amount of poison the patient consumed and whether the antidote is administered at the appropriate time, however, also affect the antidote's effectiveness. When botulinum enters the neural system, it causes conduction loss, paralyses the muscles because they are uncontrollable, and if the patient is not treated, they will have respiratory failure and eventually pass away. Also highlighted in this was the possibility of treating patients exposed to botulinum toxin A–G with a BAT product that showed excellent clinical improvement when administered early (two days after symptom onset) as opposed to late (>2 days) [20]. Total hospital length of stay (LOS) was 11.4 days for patients who received BAT product treatment early and 24.2 days for individuals who received BAT product treatment late [18]. When comparing the length of mechanical ventilation for late and early BAT product treatments, it is also noticeably five times higher [18]. The LOS was reduced most when BIG-IV (Human Botulism Immune Globulin Intravenous) was given shortly after hospital admission [21]. In the US, BIG-IV has continued to be used effectively and appropriately during the post-licensure period [21]. This evidence prove that early treatment may reduce the effects of the toxins and administration with BAT will improve recovery.

### Safe food preparation

It is of utmost importance to educate the public about the potential sources of *C. botulinum* spores and emphasise the significance of practicing proper hygiene and food safety measures to minimise the risk. It is also worth noting that the growth of *C.botulinum* and toxin production can be prevented through proper food handling, storage and preparation [2,10]. Adhering to safe food handling practices, such as promptly refrigerating perishable foods, heating foods to appropriate temperatures, and avoiding cross-contamination, can significantly reduce the risk of *C.botulinum* contamination in food [10,22]. The best way to prevent infant botulism is to keep newborns under 12 months old away from honey [2].

### Swift diagnosis is imperative

Swift recognition of the signs and symptoms of botulism is vital to ensure timely medical intervention and improved patient outcomes. Healthcare providers must receive comprehensive training to effectively identify and manage such cases because botulism is a serious neurological disorder that can be life-threatening. Because of the systemic effects, the disorder is best managed by an interprofessional team that consists of a neurologist, infectious disease expert, intensivist, pulmonologist, pharmacist, and ICU nurses.

The basic symptoms of botulism are cranial nerve palsies, which thereafter develop into symmetrical descending weakening of the trunk, extremities, and smooth muscle, culminating in flaccid paralysis [2]. A prodrome of stomach discomfort, nausea, and vomiting that starts 12 to 72 hours after ingesting the produced toxin is a common presentation of food-borne botulism. Infant botulism presents and progresses differently depending on inoculum size, host susceptibility and time to presentation [2]. Early signs often include drooling, constipation, weakness, trouble feeding, and feeble cries. When individuals exhibit bulbar symptoms and cellulitis after receiving illegal substances subcutaneously, wound botulism should be suspected. From the time spores are introduced, wound botulism takes five to 15 days to develop [2]. Only one disease, wound botulism, causes a fever. Botulism can manifest in a variety of subtle and often ignored ways, though. Since treatment should be started as soon as possible and test confirmation of botulism usually takes several days, treatment will frequently move forward based solely on clinical suspicion. Therefore, a thorough medical examination and history are crucial. Serum and stool tests for Botulinum Neurotoxin (BoNT), stool microscopy for spores, stool cultures, and wound cultures in the event of wound botulism can all be used in the laboratory to confirm the presence of botulism [2,22].

### Educational campaigns in community and further research

Awareness and outreach programmes on botulism must be implemented by local health authorities and relevant organisations. This may include disseminating educational materials to the community such as guidelines for safe feeding practices. Because Vietnam has a traditional culture for specific cuisines such as vegetarian pâté, honey, pork bologna, and fermented fish sauce, it may be possible to establish a campaign for safe food preparations.

Since the work of KF Meyer and others, various investigations have been conducted – and are still being conducted – to identify the factors that either kill or inhibit *C. botulinum* in foods. However, little is known about the fundamental biological traits of *C. botulinum* due to an overemphasis on short-term practical goals [23]. It is also imperative to conduct further research to enhance our understanding of the epidemiology, risk factors, and region-specific preventive strategies [2,23]. Nga TT and colleagues reported a case of infant botulism in a 10-month-old infant that occurred in 2021 in Hanoi to better understand the epidemiology of infant botulism in Vietnam [7]. Following this, a number of botulism cases with delayed treatment as a result of delayed diagnosis were described by Hoang LH and colleagues in 2022. Vegetarian home-canned pate was used to extract *C. botulinum* type A(B), but not stool samples. These were the first instances of foodborne botulism that have been reported in Hanoi [3].

### International collaboration

Collaborative efforts between local and international experts can foster knowledge exchange and aid in developing targeted interventions to combat botulism more effectively. In 2020, The Vietnam Ministry of Health sought WHO's support to source BAT drugs for treating patients who were infected after consuming contaminated vegetarian pâté. Some nations have antitoxin serum and other uncommon medications in their national stockpiles. Two bottles of antitoxin serum were ordered by Vietnam from Thailand for two patients. Only ten serum bottles, each costing USD 8000, were kept in Thailand's national reserve [4].

Following incidents of botulinum poisoning discovered in Ho Chi Minh City in 2023, the Drug Administration of Vietnam has negotiated with WHO on how to obtain the appropriate medications for treatment. Additionally, the Department has tasked Cho Ray Hospital to contact importers and suppliers to obtain more supplies of the vital medications. As a consequence, six vials of BAT antidote sent from the WHO’s warehouse in Switzerland arrived in the city to treat six patients [9]. In the foreseeable future, it is necessary to set up a connection between Vietnam, neighboring countries and WHO repositories for the storage of rare medicines and drugs which are in limited supply. In response to recent reports of botulism poisoning in Ho Chi Minh City, the Vietnam Ministry of Health has met with the WHO to study the organisation's storage strategy. Vietnam aims to set up three to six storage centres for 15–20 rare drugs to safeguard future infections [24].

## CONCLUSIONS

In conclusion, the escalating cases of *C. botulinum* in Vietnam recently promoted concern from health care authorities and policymakers due to the limited quantity of available BAT antitoxin. A comprehensive and multidisciplinary approach involving surveillance, public awareness, collaborative efforts, and enhanced health care resources is essential to mitigate the risks associated with botulism and protect public health in this region. Swift action in these areas can help contain the occurrence, prevent further infections, and improve patient outcomes.
